# Long-tailed macaques (*Macaca fascicularis*) can use simple heuristics but fail at drawing statistical inferences from populations to samples

**DOI:** 10.1098/rsos.181025

**Published:** 2018-09-12

**Authors:** Sarah Placì, Johanna Eckert, Hannes Rakoczy, Julia Fischer

**Affiliations:** 1 Cognitive Ethology Laboratory, German Primate Center, Kellnerweg 4, 37077 Göttingen, Germany; 2 Leibniz ScienceCampus Primate Cognition, German Primate Center, Kellnerweg 4, 37077 Göttingen, Germany; 3 Department of Developmental Psychology, University of Göttingen, Waldweg 26, 37073 Göttingen, Germany; 4 Department of Developmental and Comparative Psychology, Max Planck Institute for Evolutionary Anthropology, Deutscher Platz 6, 04103 Leipzig, Germany

**Keywords:** intuitive statistics, numerical cognition, probabilistic reasoning, comparative cognition

## Abstract

Human infants, apes and capuchin monkeys engage in intuitive statistics: they generate predictions from populations of objects to samples based on proportional information. This suggests that statistical reasoning might depend on some core knowledge that humans share with other primate species. To aid the reconstruction of the evolution of this capacity, we investigated whether intuitive statistical reasoning is also present in a species of Old World monkey. In a series of four experiments, 11 long-tailed macaques were offered different pairs of populations containing varying proportions of preferred versus neutral food items. One population always contained a higher proportion of preferred items than the other. An experimenter simultaneously drew one item out of each population, hid them in her fists and presented them to the monkeys to choose. Although some individuals performed well across most experiments, our results imply that long-tailed macaques as a group did not make statistical inferences from populations of food items to samples but rather relied on heuristics. These findings suggest that there may have been convergent evolution of this ability in New World monkeys and apes (including humans).

## 1. Introduction

The physical and social world can be described by statistical regularities: events co-occur with others repeatedly over time, resources are non-randomly distributed in space. For instance, it might rain more in some months than in others, certain fruits will be more abundant in a specific habitat, and someone's relative will repeatedly be late to a meeting while another one will always be there in case of need. In other words, frequencies of past occurrences can be informative about existing relationships between events, as well as about the likelihood of their future occurrence. Using statistical regularities to reduce uncertainty and acquire knowledge about the state of the world, what we call statistical reasoning, is key to human learning and pervades disciplines from psychology to economics, biology, physics, law and medicine [1–4]. It allows one to infer relationships between samples of observations and populations from which they stem. General knowledge can thus be induced from limited data [5], and this general knowledge can, in turn, be used to form expectations about new samples. Note that these inferences will not yield exact predictions, because of the probabilistic nature of the relation between populations and (randomly drawn) samples.

The nature and development of human statistical reasoning has long been the topic of much debate among various researchers. Some have advocated that humans become proficient in it only during later stages of childhood [6,7] and are easily prone to make errors even as adults [8,9], while others have argued that this ability emerges early on during childhood and plays an important role in structuring learning [10–12]. Whether humans explicitly understand probabilities and proficiently use statistical information is questionable. However, at an implicit level, there is now ample evidence that many aspects of statistical reasoning are already present in very young children: Preverbal infants (sometime as young as 6 months old) infer relationships between populations and samples [13], use statistical regularities to draw inferences about physical properties of objects [14] and proportions of objects to form expectations about new samples [15], as well as temporal and positional information of randomly moving objects to form expectations about which object is more likely to exit an urn [16,17]. In addition, they are sensitive to sampling processes and vary their expectations depending on whether the sampling appears to be random or not [18]. Whether children really engage in intuitive statistics and estimate probabilities, or rather rely on simpler heuristics to make predictions, is not always clear from the data [7,15,17,19,20]. Nonetheless, these results, as well as results of a study of two indigenous Mayan groups [21], show that preverbal humans and humans without formal education appear to be intuitive statisticians.

Recently, similar reasoning abilities were highlighted in non-human primates: Using a paradigm originally developed for children [15], it was shown that four species of non-human great apes [22] (hereafter apes), and one species of capuchin monkey [23] were able to use populations of food items to form expectations about sampling events. In another study, chimpanzees used proportional information to infer which of two trays containing different food/cup ratios was more likely to yield a cup containing food [24]. These findings corroborate the idea that intuitive statistics might be part of an evolutionarily more ancient core knowledge that humans share with related species [25].

Based on the same paradigm used with children [15] and apes [22], the rationale of the present study was to investigate whether long-tailed macaques (*Macaca fascicularis*), a species of Old World monkey, are able to make statistical inferences from populations of food items to samples. Previous findings with infants, apes and capuchins could point towards an evolutionarily ancient origin of this trait, given that they shared a common ancestor over 30 Ma [26]. However, as other cognitive abilities evolved in convergent fashion in capuchins and apes [27], the same might be true regarding intuitive statistics. Adding information about a species of Old World monkey will help to sharpen our knowledge of the distribution and potentially the evolutionary origin of this cognitive ability within the primate order, as the separation of the lineages between Old World monkeys and hominids happened after the separation of the lineage of New World monkeys [28].

Previous research on numerical cognition has shown that, similar to other species [29], long-tailed macaques possess the ability to differentiate between quantities [30]. Statistical reasoning goes beyond simple comparisons of quantities, in that it requires comparisons between relative frequencies (or proportions) [31] of different types of events in order to calculate the probability that an event of a given type will happen. The prerequisite ability to compare proportions has been recently highlighted in another species of macaque [32]. Additionally, similar to apes [33,34] and capuchins [35], long-tailed macaques also seem to be inclined to display inequity aversion [36], an ability necessitating a certain comprehension of relative judgement, as it requires the comparison of different outcomes obtained by different individuals, with respect to the effort they produced. In conjunction, these findings suggest that long-tailed macaques possess the prerequisites for intuitive statistics. We thus tested here whether they would perform at levels similar to those of apes and capuchins in intuitive statistical reasoning tasks.

In a series of four experiments (with seven test conditions), we presented long-tailed macaques with two transparent buckets containing populations with varying proportions of preferred (grapes) versus neutral (monkey chow) food items. Subjects watched an experimenter randomly (in appearance only) drawing a one-item sample out of each population and were given the choice between the two hidden samples. To receive a preferred food item as reward, therefore, subjects had to distinguish between the two populations in terms of their proportion of preferred to neutral food items, and use this relative frequency information to form expectations about the likely outcome of a sampling event. In Experiment 1, absolute and relative frequencies were confounded, thus, to differentiate whether monkeys really estimated probabilities, or rather relied on some simpler heuristics, several control experiments were administered. Experiments 2a–c disentangled absolute and relative frequencies of food items, while Experiment 3 controlled for the number of neutral items. If monkeys engage in statistical reasoning, they should have a preference for the samples stemming from the populations with the higher proportion of grapes. In case they rely on absolute number heuristics, they should have a preference for the hand drawing out of the populations with the higher quantity of grapes and/or the smaller quantity of monkey chow. If they do not expect any quantitative information to predict sampling events, they should have no consistent preference for either sample. Lastly, an additional control experiment checked for the use of olfactory cues (Experiment 4).

## 2. Methods

### 2.1. Subjects

Seventeen long-tailed macaques (female *N* = 8)—aged 1–10 years (table 1)—participated in this study (six of them did not reach different criteria, see electronic supplementary material for details). The monkeys lived in a large social group of 35 individuals. They were housed at the German Primate Center in Göttingen, Germany, and had access to indoor (49 m²) and outdoor areas (173 m²), which were equipped with branches, trunks, ropes and other enriching objects. All individuals were already experienced in participating in cognitive experiments and some of them previously took part in experiments requiring them to indicate a choice between two objects via pointing or reaching towards it. Tests were conducted once or twice a day between March and July 2016.

**Table 1.** List of subjects and conditions in which they participated.

name	sex	age at start of testing	participation
Ilana	f	10 years old	choice training
Paul	m	8 years old	choice training, familiarization, Exp. 1a, 1b, 2a, 2b, 3, 4
Sally	f	8 years old	choice training, familiarization, Exp. 1a, 1b, 2a, 2b, 3
Maja	f	8 years old	choice training, familiarization, Exp. 1a, 1b, 2a, 2b, 2c, 3, 4
Sophie	f	6 years old	choice training, familiarization, Exp. 1a, 1b, 2a, 2b, 3, 4
Lenny	m	6 years old	choice training, familiarization, Exp. 1a, 1b, 2a, 2b, 3, 4
Isaak	m	4 years old	choice training, familiarization
Mila	f	3 years old	choice training, familiarization, Exp. 1a, 1b, 2a, 2b, 3, 4
Ilia	m	3 years old	choice training, familiarization, Exp. 1a, 1b, 2a, 2b, 3, 4
Linus	m	3 years old	choice training, familiarization, Exp. 1a, 1b, 2a, 2b, 3, 4
Max	m	3 years old	choice training, familiarization, Exp. 1a, 1b, 2a, 2b, 3, 4
Snickers	m	2 years old	choice training, familiarization
Mars	m	2 years old	choice training, familiarization, Exp. 1a, 1b, 2a, 2b, 3, 4
Lord	m	2 years old	choice training, familiarization, Exp. 1a, 1b, 2a, 2b, 3, 4
Sissi	f	2 years old	choice training, familiarization
Milka	f	1 year old	choice training, familiarization
Sambia	f	1 year old	choice training, familiarization

### 2.2. Experimental set-up

**Figure 1.**
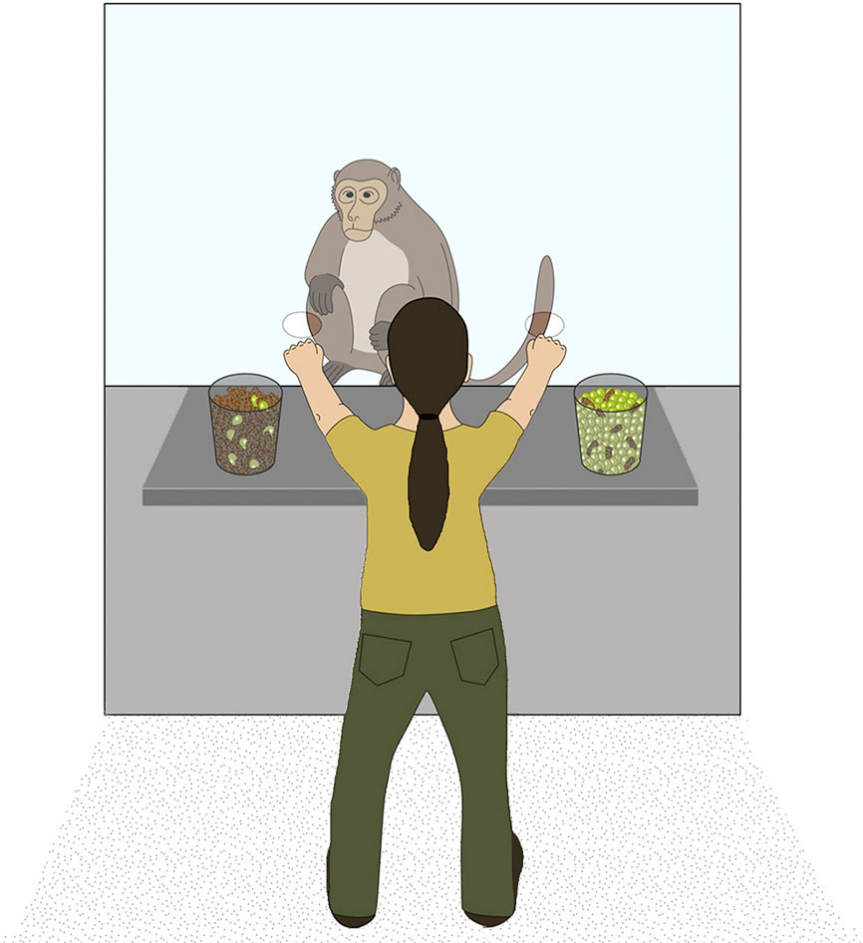
Experimental set-up. The monkey observed the experimenter drawing two hidden samples out of two populations of food items. Subsequently, the subject was given the choice between the two hidden samples. © MPI for Evolutionary Anthropology.

The testing cage (260 × 225 × 125 cm; height × width × depth) was adjacent to the indoor enclosure, and could be subdivided into six experimental compartments. Subjects were tested individually in one compartment (105 × 110 cm; height × length) to which an attachable cage (73 × 53 × 35 cm; height × width × depth) was fixed, allowing subjects to have better access to the experiment. The cage was built in metallic mesh, except for the front part that separated the monkey and the experimenter, which consisted in a removable Plexiglas pane (27 × 34 cm; height × length). The pane had two small holes (diameter of each hole: 3.5 cm; distance between holes: 27 cm; figure 1) through which subjects could insert their arm to indicate a choice. The experimenter stood behind a wheeled table (85 × 80 × 50 cm; height × width × depth) that was set in front of the cage, on which the stimuli were presented.

### 2.3. Study design and procedure

The study comprised four experiments (seven test conditions) preceded by a short choice training, a preference test and a familiarization phase (see electronic supplementary material for a description). Each test condition consisted of 12 test trials, evenly divided into two sessions. Test sessions always started with an additional preference test to make sure that monkeys’ preference for one of the two food types was consistent over time. Twice in a row, subjects were given the choice between one grape and one piece of monkey chow. Hence, each session consisted of two preference trials and six test trials. In each test trial, subjects were confronted with two populations consisting of a pre-determined mix of grapes (preferred food type) and monkey chow (neutral food type) contained in transparent buckets. Items of the two food types had the same approximate size but differed in colour so as to render them easily distinguishable one from the other. The experimenter presented both populations on a table, shook them one after the other (always starting with the one on the right-hand side) and tilted them slightly forward to give the subject a good overview. She then closed her eyes, reached into the buckets and simultaneously drew one item (always the majority type, except in Experiment 3) out of each bucket in a way that kept the item hidden from the subject. While keeping the food concealed in her fists, the experimenter subsequently moved both hands towards the holes and allowed the monkey to make a choice (figure 1). Once the subject had touched one of the hands, it received its content as a reward. Before the next trial started, the experimenter refilled the buckets out of sight of the subject and placed them back on the table. The position of both populations was counterbalanced across trials and subjects. To make sure that subjects chose between the samples and not between the two buckets standing on the table, the experimenter crossed her arms in half of the trials before allowing the monkey to indicate a choice. Trials with and without crossing were alternated. Subjects were thus required to conclude from the information provided by the populations, which fist was more likely to contain a preferred food item as a sample. The position of the favourable sample was counterbalanced across trials (but see Experiment 1a).

### 2.4. Coding procedure

Every session was video-recorded. The experimenter coded monkeys' choices live: Whenever monkeys chose the sample stemming from the favourable population, she coded it as a success on a paper sheet, and as a failure when they chose the alternative option. A second blind observer coded 25% of the sessions, using the video recordings. Agreement between the experimenter and the second coder was perfect for all experiments (100% agreement).

### 2.5. Data analysis

To test whether monkeys’ mean group performance for each experiment was different from chance, we computed one sample two-tailed *t*-tests. To analyse individual performances, we used binomial tests to calculate the probability to observe the number of successes or any higher number out of 12 trials, conditional on an underlying probability of success in one trial equal to 0.5. To adjust for an inflation of the family-wise significance level, we report *p*-values adjusted for multiple testing [37]. In addition, we modelled monkeys' performance conditional on whether arms were crossed or not, using a generalized linear mixed model, with binomially distributed response and logit link function. The fixed effect was the two different states of the hands (crossed or not crossed). To check whether the monkeys (at the group level) learnt to associate a proportion with the preferred reward, we computed the correlation between group performance and trial number separately for all conditions. All these analyses were performed using R [38].

## 3. Results and discussion

### 3.1. Experiment 1a and b

In Experiment 1, we wanted to investigate whether long-tailed macaques use quantitative information to make inferences about sampling events. As Experiment 1a was not fully randomized with regard to the side on which the favourable sample appeared (for some individuals, the favourable sample appeared more often in one hand than in the other, which might have favoured side-biased individuals), we added Experiment 1b to our study, which was corrected for this flaw. We used the same proportions of grapes and monkey chow in both experiments, but varied the absolute quantities of food items. Eleven subjects participated (table 1). Both buckets contained the same number of food items in each experiment (the total number of items was 80 in both buckets of Exp. 1a and 250 in Exp. 1b), with a distribution of grapes to monkey chow of 4 : 1 in one bucket and 1 : 4 in the other (table 2).

**Table 2.** Individual performance in each of the seven conditions. The proportions of grapes to monkey chow items for each population (Favourable population versus less favourable population, grapes : monkey chow) are included for each condition. For each condition and each individual, we report the sum of correct choices (in brackets: number of times they chose the right side) within the 12 trials.

individual	Exp. 1a 64 : 16 versus 16 : 64	Exp. 1b 200 : 50 versus 50 : 200	Exp. 2a 12 : 3 versus 100 : 400	Exp. 2b 48 : 12 versus 48 : 192	Exp. 2c 48 : 12 versus 48 : 192	Exp. 3 128 : 160 versus 8 : 160	Exp. 4 64 : 16 versus 16 : 64
Paul	7 (6)	6 (4)	6 (2)	6 (2)	/	7 (7)	7 (5)
Sally	9 (9)	9 (9)	5 (11^a^)	10 (6)	/	8 (4)	/
Maja	9 (7)	11^a^ (5)	5 (7)	12^a^ (6)	10 (4)	12^a^ (6)	5 (9)
Sophie	8 (8)	7 (11^a^)	7 (9)	5 (11^a^)	/	7 (11^a^)	7 (9)
Lenny	6 (6)	6 (6)	6 (6)	5 (4)	/	6 (6)	6 (6)
Mila	11^a^ (3)	7 (3)	8 (2)	6 (8)	/	8 (8)	7 (5)
Ilia	6 (6)	6 (4)	5 (4)	7 (7)	/	8 (4)	4 (4)
Linus	6 (6)	7 (5)	6 (6)	6 (6)	/	6 (6)	5 (5)
Max	9 (1^a^)	6 (0^a^)	9 (3)	6 (0^a^)	/	6 (6)	6 (0^a^)
Mars	8 (4)	7 (5)	8 (6)	7 (7)	/	4 (6)	6 (6)
Lord	5 (5)	7 (5)	8 (6)	8 (10)	/	8 (8)	6 (12^a^)

^a^Indicates performances and side selectivity that were statistically above chance.

**Figure 2.**
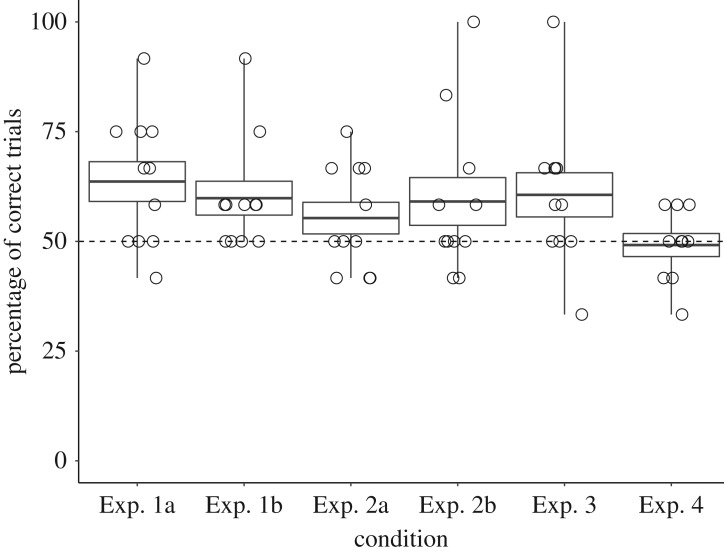
Group performance across conditions. The boxplots represent the mean percentage of trials (±1 s.e. as well as the maxima and minima) in which subjects selected the favourable sample. White circles represent individual data.

On average, subjects selected the sample drawn out of the favourable population in 7.64 trials (63.6%; s.d. *=* 1.8 trials) in Exp. 1a and in 7.18 trials (59.8%; s.d. = 1.54 trials) in Exp. 1b (figure 2), significantly more frequently than expected by chance (Exp. 1a: *t*(10) = 3.01, *p* = 0.01, *d* = 0.91; Exp. 1b: *t*(10) = 2.55, *p* = 0.03, *d* = 0.77). At the individual level, only two individuals (Mila and Maja) performed significantly above chance (Mila in Exp. 1a: *p* = 0.02, Maja in Exp. 1b: *p* = 0.02). This suggests that, as a group, long-tailed macaques use some quantitative information (absolute and/or relative) to draw inferences from population to sample.

### 3.2. Experiment 2a, b and c

As absolute and relative frequencies of preferred versus neutral food items were confounded in Experiment 1, it is not possible to distinguish between inferences based on relative frequencies (choosing the sample with the higher probability of being a preferred food item) from merely relying on absolute frequencies (choosing the sample drawn from the population with absolutely more preferred food items). As such, we added Experiment 2a–c to differentiate between these possibilities. We disentangled absolute and relative frequencies of grapes in two ways: In Experiment 2a, the absolute quantity of grapes was higher in the population with the less favourable proportion of grapes to monkey chow (table 2). If monkeys based their choice on the absolute quantity of preferred food items, we expected them to perform below chance level in this condition. In Experiment 2b, the absolute quantity of grapes was the same in both populations and therefore inconclusive (table 2). Hence, if subjects relied on absolute numbers, we expected them to choose both populations at similar rates. If, however, they used proportional information to solve the task, they should succeed in both Experiment 2a and 2b. Subjects that were successful in Experiment 2b were tested in a third condition, Experiment 2c. Experiment 2c was designed as a follow-up condition on Experiment 2b, to make sure that subjects recognized that both buckets contained the same absolute quantity of preferred food items. The higher quantity of neutral food items in one of the populations in Experiment 2b might have led to a visual appearance of fewer grapes in this bucket. This could allow the simple heuristic of choosing the sample from the bucket with a higher visible number of grapes. To shed light on that, we used the same quantities of food as in Experiment 2b, but this time filled the two food types in the buckets one after the other in the presence of the monkey, thereby ensuring that subjects were aware of both buckets containing the same amount of grapes. All eleven subjects participated in both Experiment 2a and 2b. Only Maja participated in Experiment 2c (table 1).

On average, subjects selected the sample drawn out of the favourable population in 6.64 trials (55.3%; s.d. = 1.43 trials) and 7.09 trials (59.1%; s.d. = 2.17) in Exp. 2a and Exp. 2b, respectively, which was not different from chance (Exp. 2a: *t*(10) = 1.47, *p* = 0.17, *d* = 0.44; Exp. 2b: *t*(10) = 1.67, *p* = 0.13, *d* = 0.5). At the individual level, only Maja was significantly above chance in Exp. 2b (*p* < 0.01), while Sally's performance, with 10 out of 12 correct trials, was also good (table 2). Maja's performance was not above chance in Exp. 2c after correction for multiple testing, but still good with 10 correct trials (table 2). Taken together, Experiments 2a–c suggest that Maja and Sally did not engage in intuitive statistical inferences when widely varying numbers of grapes were used (Exp. 2a), while they seem to have done so when the quantity of grapes was kept constant (Exp. 2b and 2c for Maja).

### 3.3. Experiment 3

The aim of this experiment was to rule out another potential alternative explanation of the patterns of results in Exp. 1. Successful performance in this experiment could have been due to a much simpler strategy of avoiding (samples from) the population with the higher absolute number of neutral food items. In Experiment 3, therefore, both populations contained the same absolute number of monkey chow pieces but different amounts of grapes (table 2). Hence, we expected monkeys to perform at chance level in this experiment in the case that they relied on absolute numbers of monkey chow pellets to make their decisions. If, however, they took into account the proportion or the absolute quantity of grapes, they should succeed in this task. All eleven subjects participated (table 1). The procedure was the same as in Experiment 1, with the following exception: To maintain the appearance of random sampling, choosing the ‘correct’ population did not always result in a grape as sample. Instead, sampling was proportional, i.e. the sample of the favourable population was a grape in 5 out of 12 trials, with the same rewarding pattern maintained between individuals. The sample of the less favourable population was a grape every 24 trials, meaning that half of the monkeys were never rewarded with a grape stemming from the less favourable population. Additionally, this experiment allowed us to investigate whether monkeys would distinguish between a correct choice and a favourable outcome [20]. A correct choice can result in an unfavourable draw because of the laws of probability. For this reason, subject should stick to choosing the sample drawn out of the favourable population and not change their choices according to past outcomes. If monkeys were aware of this distinction, we predicted that their choices would be consistent throughout the 12 trials of the experiment, despite receiving neutral items as rewards for correct choices.

On average, subjects selected the sample drawn out of the favourable population in 7.27 trials (60.6%; s.d. = 2.00 trials), not significantly different from chance (*t*(10) = 2.11, *p* = 0.06, *d* = 0.64). At the individual level, only Maja was significantly above chance (*p* < 0.01). These results suggest that at least Maja was not relying on the absolute number of neutral items to make her inferences. Her choice was consistent across the 12 trials of the experiment, suggesting that it was not affected by the rewarding pattern.

### 3.4. Experiment 4

In the previous experiments, it could not be excluded that subjects solved the task by means of olfactory cues. Experiment 4 therefore served as a control condition to rule this possibility out. Ten subjects participated (table 1). We used the same populations as in Experiment 1a (64 : 16 versus 16 : 64) and the same procedure as in Experiment 1b, but both buckets were concealed by two opaque occluders, preventing subjects from seeing their content.

On average, subjects selected the sample drawn out of the favourable population in 5.9 trials, (49.2%; s.d. = 0.99 trials), not significantly different from chance (*t*(9) = −0.32, *p* = 0.76, *d* = 0.10; no individual performance was above chance). This suggests that none of our subjects based their decisions on olfactory cues.

## 4. Possible confounding effects (Experiments 1–4)

Our results indicated no main effect of arms' positioning, whether they were straight or crossed (estimate ± s.e. = 0.01 ± 0.15, *z* = 0.07, *p* = 0.94). Furthermore, in every condition there was no significant correlation between group performance and trial number (see electronic supplementary material, table S2 and figure S1).

## 5. General discussion

The present study tested for intuitive statistics in long-tailed macaques by probing their ability to draw inferences from populations of food items to samples randomly (in appearance) drawn from the populations. The main results were the following: As a group, long-tailed macaques performed above chance level only in the first experiment in which absolute and relative quantities were confounded, but did not perform above chance level in the experiments in which these quantities were deconfounded (Exp. 2 and 3). Comparing absolute quantities of grapes could have been sufficient to select the favourable sample above chance level in Experiment 1. By contrast, human infants, apes and capuchins performed above chance level in experiments in which absolute and relative quantities of the preferred items were deconfounded. Our results demonstrate differences in statistical reasoning abilities between long-tailed macaques on the one hand, and 12-month-old children [15,39], apes [22] and capuchins [23] on the other hand. This pattern suggests a convergent evolution of this ability in apes and capuchins.

When focusing on individual performance rather than on group means, a different picture emerged. Although most individuals performed at chance level, two adult females, Maja and Sally, performed well in all conditions testing for statistical reasoning, except in Experiment 2a, in which absolute and relative frequencies of preferred items were deconfounded. There are two possible and mutually exclusive explanations for this pattern of behaviour. The first one is that in Experiment 2a, the bucket with the less favourable proportion contained a much larger number of grapes. Thus, it took not only the ability to compare proportions to solve this task but also the ability to inhibit the choice of the larger amount of preferred food, which is a highly salient stimulus with the potential to interfere in rational decision-making [40–42]. This task might have been harder for the long-tailed macaques than for the other species, as they seem to have more difficulties at inhibiting their impulses [43,44]. For this reason, we added Experiment 2b, in which we equalled the absolute number of grapes of both populations. Both adult females performed well in this condition, which indicates that when inhibiting the choice of the larger amount of preferred food is not an issue any more, they can use proportions to draw statistical inferences.

The second explanation for Maja and Sally's performance results directly from a possible confound within this bucket paradigm (and might thus also be present in previous studies with human children, apes and capuchins): In all conditions except Experiment 2a, the higher proportion of grapes was confounded with the higher quantity of visible grapes. Subjects could have succeeded in most conditions by selecting the sample drawn out of the population with the higher number of visible grapes, without taking into account the grapes hidden by monkey chow items and thus without considering proportions at all (see electronic supplementary material, figure S2 for pictures of the different populations). In Experiment 2a, the difference between the quantities of visible grapes of both populations was less striking, which might explain why Maja and Sally performed at chance level. To rule out this alternative explanation, we added Exp. 2c. In this condition, the grapes were added to the buckets before the monkey chow so that Maja could have access to the complete information. Her performance in this experiment, even if not perfect, was still high (10 correct trials out of 12) and suggests that she relied on proportions. To better decide between these alternatives, future studies should make sure to work with transparent populations, i.e. populations in which all items are visible at all times. Performance of previously tested species should maybe also be reassessed using appropriate controls, so as to definitely rule out this alternative explanation.

Another improvement to this paradigm would be to switch from deterministic rewarding to probabilistic rewarding in all experiments. In the initial paradigm [15] tested on children, as well as in the ape study [22], the rewarding pattern was always certain. It was thus not possible to assess whether subjects distinguished between a correct choice and a favourable draw. In Experiment 3, in which we deconfounded the relative and absolute number of neutral items, we also changed the certain rewarding to a probabilistic one to address this question. It seems that Maja was aware of the difference between a correct choice and a favourable draw because despite her receiving neutral food items as reward she kept choosing the correct sample rather than varying her choices depending on the distribution of past outcomes.

Why did these two females, but especially Maja, perform better than any other monkey in this study? The reason is difficult to pin down as there might be several factors at play and as we did not make any specific prediction about this individual variation. It might have to do with variation in motivation and sustained attention rather than ability, as Maja in particular always seemed calm and attentive when being tested, while many of the other subjects often looked or momentarily moved away. We did always wait for our subjects to pay attention to the testing material before proceeding with a trial, but this looking and moving away might suggest some lack of interest in the experiment. For the sake of comparability with previous studies, we did not counterbalance the order of the conditions, to control for order effects on performance. We thus cannot rule out that group performance was better in Exp. 1 only because monkeys were more motivated in the beginning compared to the end of the study. Another less observable factor might be a variation in how monkeys interpreted the drawing, as inferences based on proportions should only have been made under the assumption that the drawing was blind. Some monkeys might not have made this assumption even if the experimenter closed her eyes during the drawing, either because they considered the human experimenter omniscient and almighty (not too unreasonable from their everyday experience), or because they assumed that she could haptically distinguish monkey chow from grapes during the drawing process. To address this concern, future studies could integrate a mechanical device performing the drawing into the paradigm. Finally, a pre-existing side-bias, or an impulse to choose whatever side on which preferred food was displayed, might have prevented our subjects from engaging in more sophisticated decision-making processes. In fact, several individuals were consistently side-biased across conditions (e.g. Sophie and Max, table 2), but whether these side-biases are causes of their chance performance, or consequences of their inability to understand the task is not clear.

Importantly, although our monkeys as a group performed worse than the other species in the experiment in which absolute and relative number of preferred items were deconfounded (Exp. 2), when looking at individual performances, similar patterns emerge between species. For example, only one of the capuchins (see Kato in the online resources of the capuchin study [23]), and none of the orang-utans (see the performance of orang-utans in the online resources of the apes study [22]) were above chance in all experiments. If we want to draw firm conclusions about the use of statistical reasoning, we need to consider individual performance and consistency across all conditions. Analyses at the group level might obfuscate the possibility that individuals relied on different quantity-based heuristics interchangeably, a strategy already described in school-aged children [20]. Additionally, it has to be noted that capuchins as a group failed to draw correct inferences in the task in which the absolute number of neutral items was kept constant (see Experiment 4 of the capuchin study). Thus, it cannot yet be ruled out that capuchins solved the different tasks by avoiding the sample drawn out of the population with more neutral items. In the children study, the methods of the experiments that were meant to rule out this quantity heuristic (Experiments 3 and 4 of the children study) are ambiguous. In fact, a third type of objects, with which children did not have any prior experience, was added to the populations. The authors assumed that children considered these objects as neutral and thus did not try to avoid the populations containing more of them. However, by making the opposite assumption, results of both experiments would suggest that children did avoid the population with a higher quantity of this third type of objects. So far, only apes were recently tested in such a task and performed well [45].

In summary, at the group level, our subjects' performance did not match the capacities described in human children, great apes and capuchins. It remains an open question whether this observed difference was due to performance limitations such as a lack of sustained attention and motivation or to a failure to interpret the drawing as random, or due to a true competence limitation in their statistical reasoning capability. We found some evidence that two individuals used proportions to solve the tasks. However, this is not sufficient to generalize to the group level. Our findings, together with findings of studies with human children, apes and capuchins, suggest that there might have been convergent evolution of intuitive statistics (or any of the prerequisite components for this ability) in New World monkeys and apes, at least in a context of food choice. However, before reaching any sharp conclusion about the evolutionary origins of statistical reasoning, appropriate controls as well as analyses of individual performances should be added to previous studies to rule out the use of quantity-heuristics that could be involved in such decision-making and to rule out possible confounds that might also account for the data. This was not done thoroughly enough until now. Furthermore, more species of Old and New World monkey should be tested for intuitive statistics to help make precise any claim about the evolutionary origins of this ability.

## Supplementary Material

Training, familiarization, tables ans figures

## Supplementary Material

Populations; Exp1a; Exp2c; Exp4

## Data accessibility

Supporting material has been uploaded as part of the electronic supplementary material. The datasets generated and analysed during the current study, as well as the R scripts used for the analyses, have been deposited with Dryad Digital Repository: (http://dx.doi.org/10.5061/dryad.kp275f9) [46].
