# Supplementary material for: Long-tailed macaques (Macaca fascicularis) can use simple heuristics but fail at drawing statistical inferences from populations to samples
Source: R Soc Open Sci. 2018 Sep 12;5(9):181025. doi: 10.1098/rsos.181025 (PMC6170548; doi:10.1098/rsos.181025)
Supplement: Training, familiarization, tables ans figures [file rsos181025supp1.docx]

Title: Long-tailed macaques (*Macaca fascicularis*) can use simple heuristics but fail at drawing statistical inferences from populations to samples

Sarah Placì^1,2,3*^, Johanna Eckert^2,3,4^, Hannes Rakoczy^2,3+^, Julia Fischer^1,3+^

^1^Cognitive Ethology Laboratory, German Primate Center, Kellnerweg 4, 37077 Göttingen, Germany

^2^Department of Developmental Psychology, University of Göttingen, Waldweg 26, 37073 Göttingen, Germany

^3^Leibniz-ScienceCampus Primate Cognition, German Primate Center, Kellnerweg 4, 37077 Göttingen, Germany

^4^Department of Developmental and Comparative Psychology, Max Planck Institute for Evolutionary Anthropology, Deutscher Platz 6, 04103 Leipzig, Germany

*Corresponding Author: Sarah Placì

^+^Equal contribution

Email: [splaci@dpz.eu](mailto:splaci@dpz.eu)

Phone +49 551 3851-0

Supplementary material

*1. Choice Training*

As some of the younger monkeys had never before participated in a choice paradigm, all subjects went through a choice training phase. In the first session (consisting of six trials), the experimenter held one grape in one of her hands (left and right hand were counterbalanced), and nothing in the other hand. She showed the two upwards-facing open hands to the subject, then closed them and held the two fists close to the holes. As soon as the monkey touched one of the two fists, the experimenter opened it and, if it contained any food, handed it to the monkey. The procedure of the second session of choice training was exactly the same, but this time the experimenter crossed her arms before approaching the two holes, so that the monkey was required to track the movement of hands. The following sessions were all similar to the second one. Subjects received choice training sessions until they reached the criterion of 5/6 correct choices (i.e. choosing the hand containing a grape) within one session (starting with session 2). If criterion was reached, monkeys proceeded to the familiarization phase. Fifteen individuals had reached criterion until the end of the study and were allowed to take part in the familiarization phase (see Supplementary Table S1 for a summary of the number of sessions needed for each monkey before reaching criterion).

*2. Preference Test*

All monkeys who reached criterion in the Choice Training also went through a preference testing phase, to make sure that they had a consistent preference for grapes over monkey chow (except Maja who started later and for whom we decided that the preference test before each experiment was sufficient). In each session, the experimenter held one grape in one hand and one monkey chow item in the other, showed them to the monkey, then closed her fists and approached them to the holes in the Plexiglas. Each session consisted of six trials. Criterion for a consistent preference was to choose grapes 5 times over 6 within one session. All subjects chose grapes 6 times over 6 in the first session, except Lord who once chose a monkey chow item.

*3. Familiarization*

To familiarize monkeys with the procedure and the process of drawing, they underwent a familiarization phase before proceeding to the experiments. Subjects were presented with two transparent buckets, one containing 100 grapes, the other containing the same amount of monkey chow. The experimenter simultaneously drew one item from each of the two buckets, kept them hidden in her fists and held the closed hands in front of the holes to allow the subject to come within reach of them. Once the subject had made its choice by touching one of the hands, the experimenter showed the content of the chosen fist and gave the food item to the monkey. In half of the trials, the experimenter crossed her arms after the drawing process, before the subject indicated a choice. This was to ensure that monkeys did not simply make a choice between the two buckets, but instead had to track the sampling process and the movement of the hands containing the samples. Trials with and without crossing were alternated. Subjects received familiarization sessions until they reached the criterion of 10/12 correct choices (i.e. choosing the hand containing a grape) within two consecutive sessions. Eleven individuals reached criterion and were subsequently allowed to take part in the following experiments (see Supplementary Table S1 for a summary of the number of sessions needed for each monkey before reaching criterion).

**Supplementary Table S1.** Number of session needed for each subject before reaching criterion during the choice training and the familiarization phase.

| **Individual** | **Phase** | **N° Session** |
| --- | --- | --- |
| Ilana | Choice training | Did not reach criterion |
| Paul | Choice training | 2 |
| Sally | Choice training | 3 |
| Maja | Choice training | 2 |
| Sophie | Choice training | 2 |
| Lenny | Choice training | 2 |
| Isaak | Choice training | 2 |
| Mila | Choice training | 6 |
| Ilia | Choice training | 2 |
| Linus | Choice training | 4 |
| Max | Choice training | 2 |
| Snickers | Choice training | 5 |
| Mars | Choice training | 7 |
| Lord | Choice training | 4 |
| Sissi | Choice training | 2 |
| Milka | Choice training | Did not reach criterion |
| Sambia | Choice training | Did not reach criterion |
| Paul | Familiarization | 2 |
| Sally | Familiarization | 4 |
| Maja | Familiarization | 2 |
| Sophie | Familiarization | 2 |
| Lenny | Familiarization | 2 |
| Isaak | Familiarization | Did not reach criterion |
| Mila | Familiarization | 3 |
| Ilia | Familiarization | 4 |
| Linus | Familiarization | 2 |
| Max | Familiarization | 6 |
| Snickers | Familiarization | Did not reach criterion |
| Mars | Familiarization | 6 |
| Lord | Familiarization | 5 |
| Sissi | Familiarization | Did not reach criterion |

**Supplementary Table S2.** Group performance in each trial. For each trial and each condition, we calculated the group’ mean percentage of selecting the favorable sample.

|  | Exp. 1a | Exp. 1b | Exp. 2a | Exp. 2b | Exp. 3 | Exp. 4 |
| --- | --- | --- | --- | --- | --- | --- |
| Trial 1 | 63.6 | 45.5 | 36.4 | 27.3 | 90.9 | 70 |
| Trial 2 | 72.7 | 27.3 | 72.7 | 54.5 | 36.4 | 30 |
| Trial 3 | 27.3 | 54.5 | 63.6 | 72.7 | 27.3 | 30 |
| Trial 4 | 81.8 | 54.5 | 54.5 | 72.7 | 45.5 | 100 |
| Trial 5 | 72.7 | 72.7 | 54.5 | 63.6 | 63.6 | 50 |
| Trial 6 | 54.5 | 100 | 45.5 | 27.3 | 72.7 | 50 |
| Trial 7 | 63.6 | 81.8 | 54.5 | 81.8 | 63.6 | 60 |
| Trial 8 | 63.6 | 72.7 | 54.5 | 54.5 | 100 | 50 |
| Trial 9 | 81.8 | 36.4 | 36.4 | 54.5 | 81.8 | 60 |
| Trial 10 | 63.6 | 54.5 | 45.5 | 63.6 | 100 | 30 |
| Trial 11 | 54.5 | 54.5 | 81.8 | 72.7 | 27.3 | 50 |
| Trial 12 | 63.6 | 63.6 | 63.6 | 63.6 | 18.2 | 10 |

**Supplementry Fig. S1**. Correlation between group performance and trial number for each condition. The dark lines are regression lines and the light lines show the closest fit to the data points.
