# Supplementary material for: Long-tailed macaques (Macaca fascicularis) can use simple heuristics but fail at drawing statistical inferences from populations to samples
Source: R Soc Open Sci. 2018 Sep 12;5(9):181025. doi: 10.1098/rsos.181025 (PMC6170548; doi:10.1098/rsos.181025)
Supplement: Populations; Exp1a; Exp2c; Exp4 [file rsos181025supp2.docx]

Title: Long-tailed macaques (*Macaca fascicularis*) can use simple heuristics but fail at drawing statistical inferences from populations to samples

Sarah Placì^1,2,3*^, Johanna Eckert^2,3,4^, Hannes Rakoczy^2,3+^, Julia Fischer^1,3+^

^1^Cognitive Ethology Laboratory, German Primate Center, Kellnerweg 4, 37077 Göttingen, Germany

^2^Department of Developmental Psychology, University of Göttingen, Waldweg 26, 37073 Göttingen, Germany

^3^Leibniz-ScienceCampus Primate Cognition, German Primate Center, Kellnerweg 4, 37077 Göttingen, Germany

^4^Department of Developmental and Comparative Psychology, Max Planck Institute for Evolutionary Anthropology, Deutscher Platz 6, 04103 Leipzig, Germany

*Corresponding Author: Sarah Placì

^+^Equal contribution

Email: [splaci@dpz.eu](mailto:splaci@dpz.eu)

Phone +49 551 3851-0

Supplementary material

**Experiment 1a**


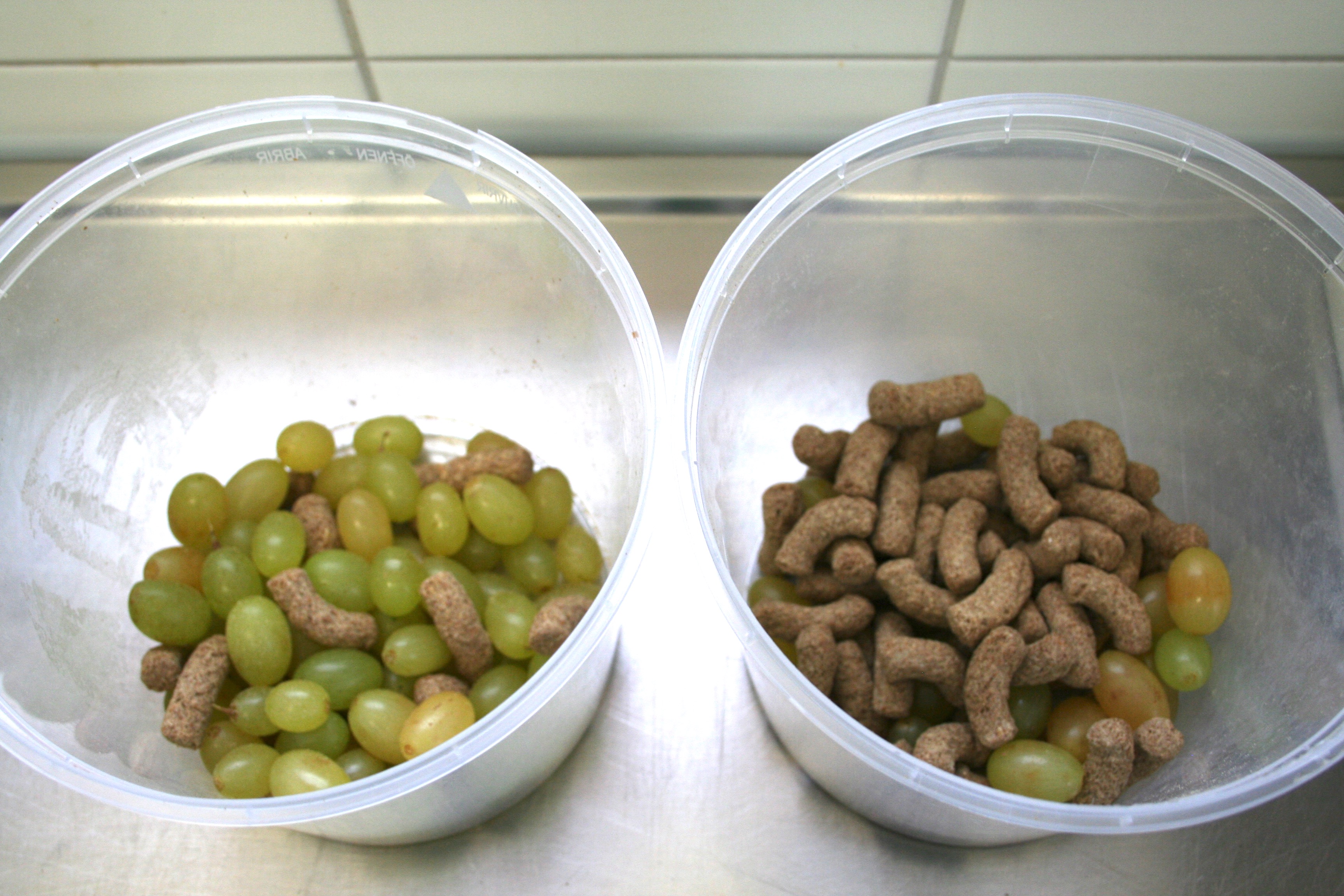

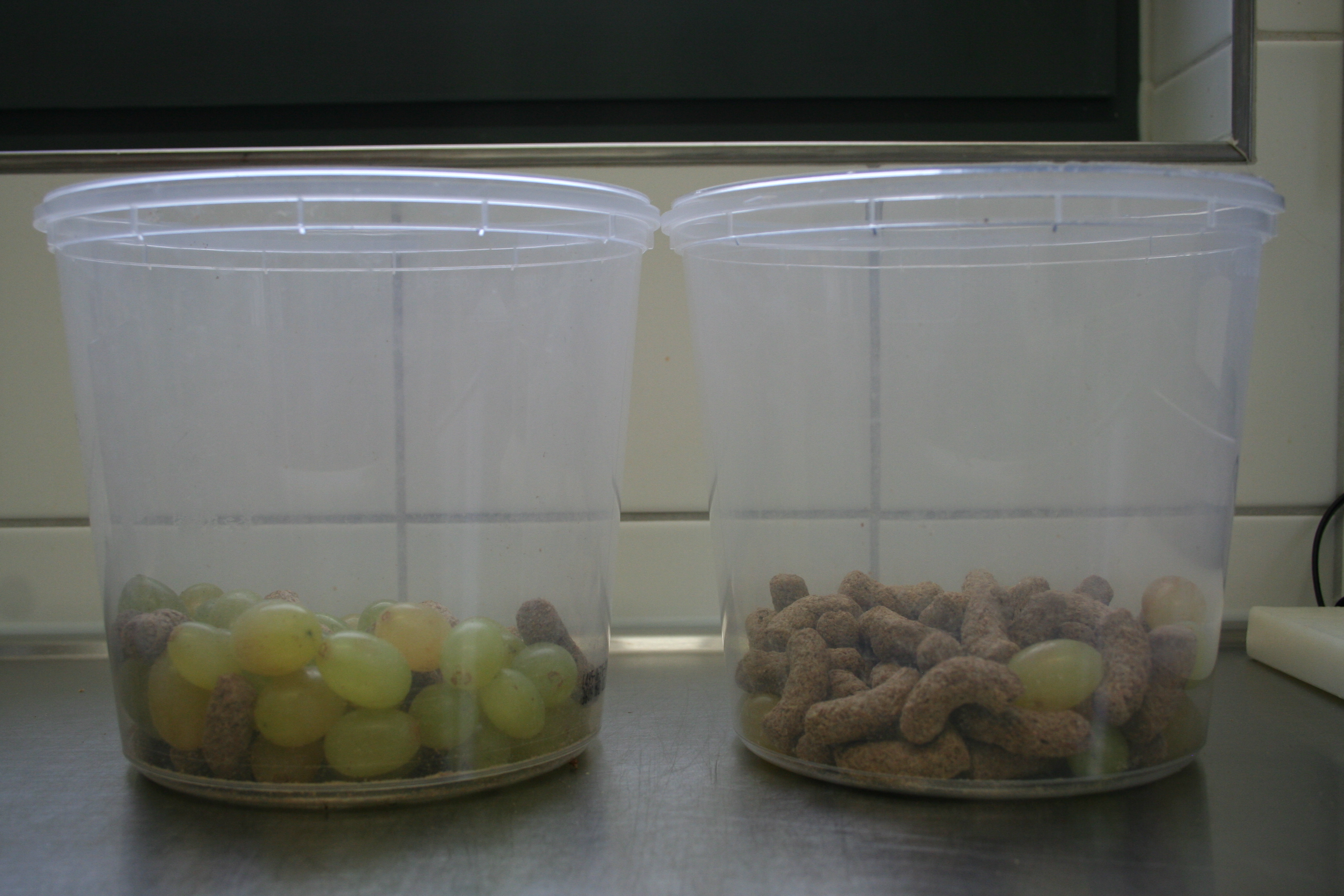


**Experiment 1b**

**
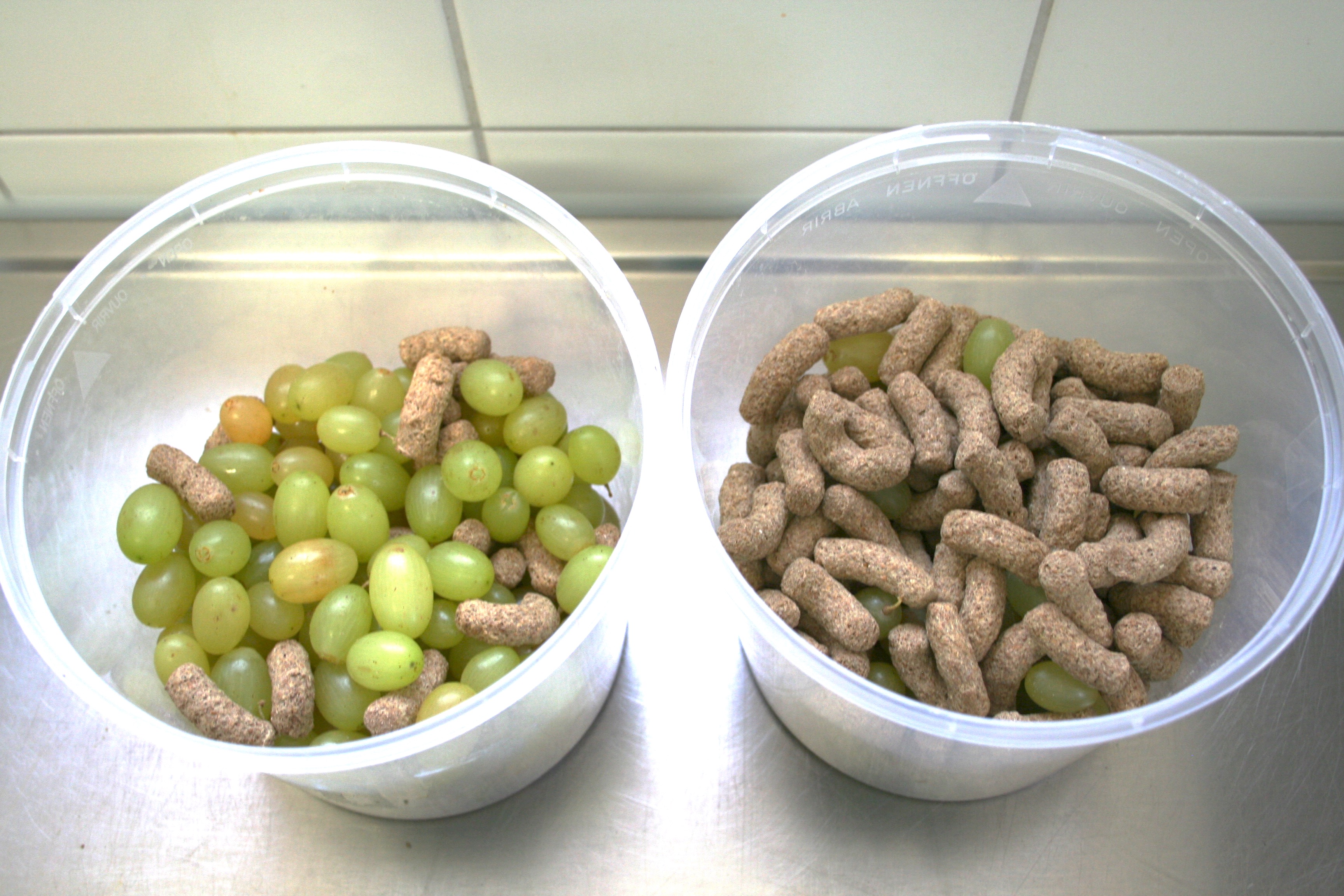

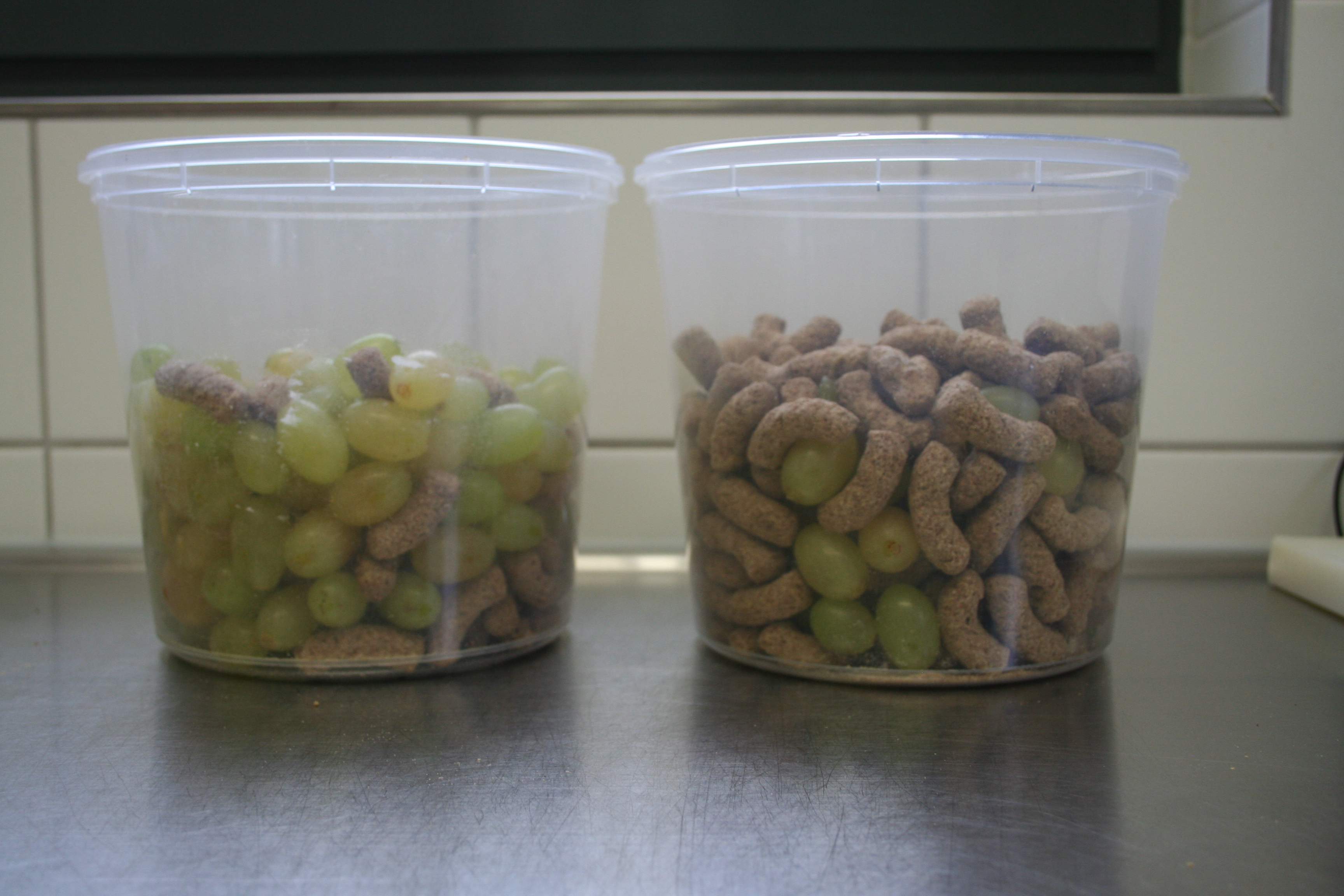
**

**Experiment 2a**

**
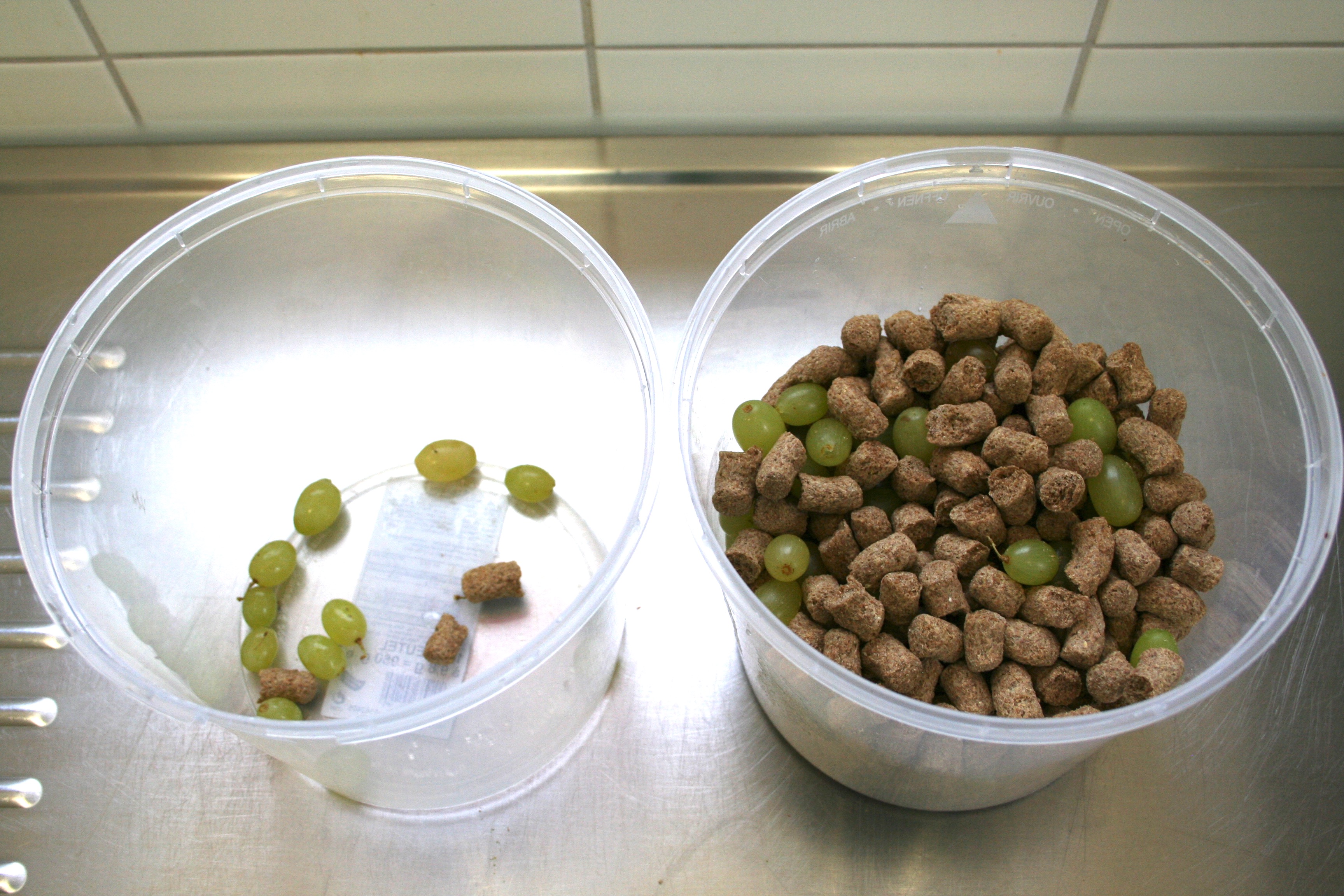

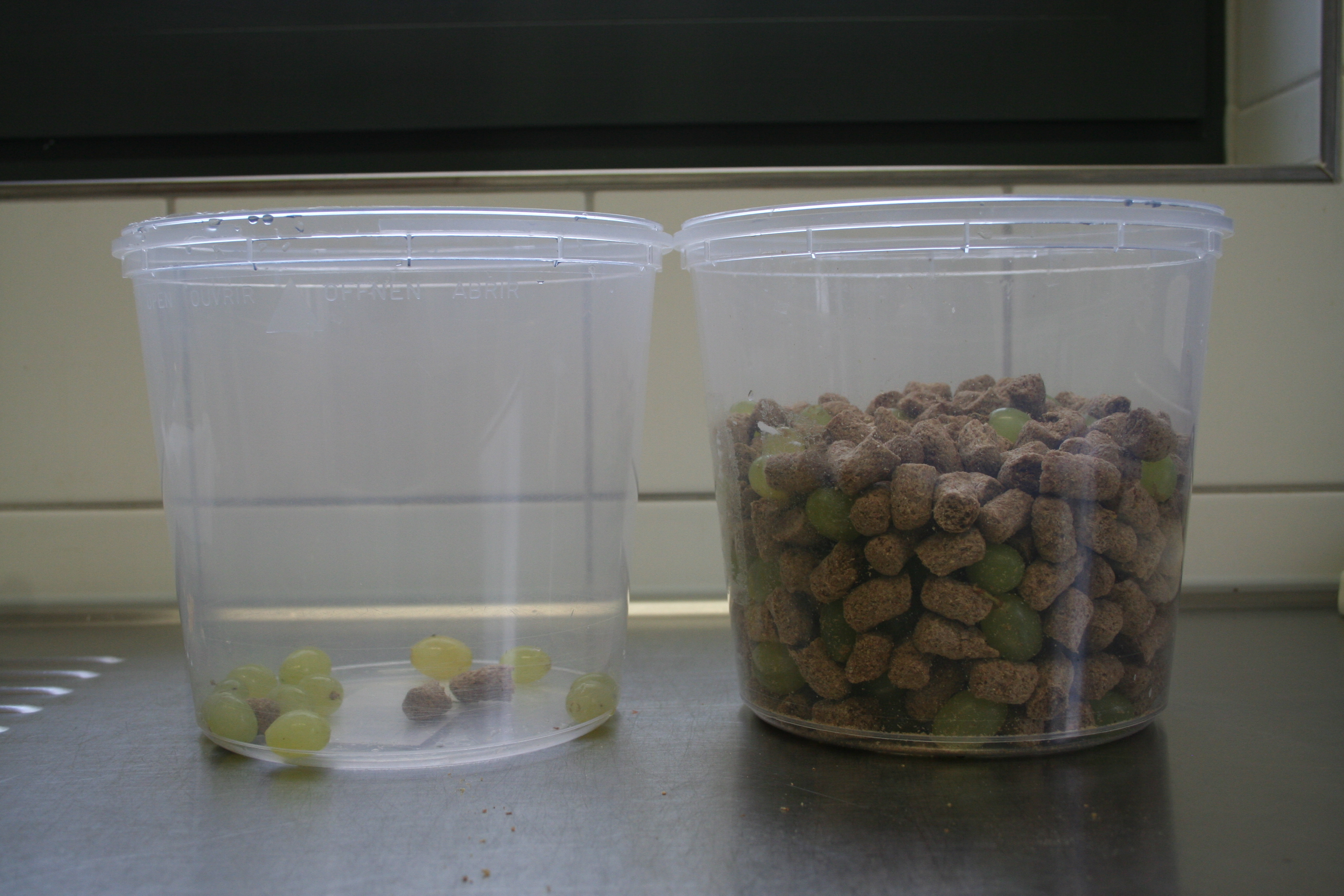
**

**Experiment 2b**

**
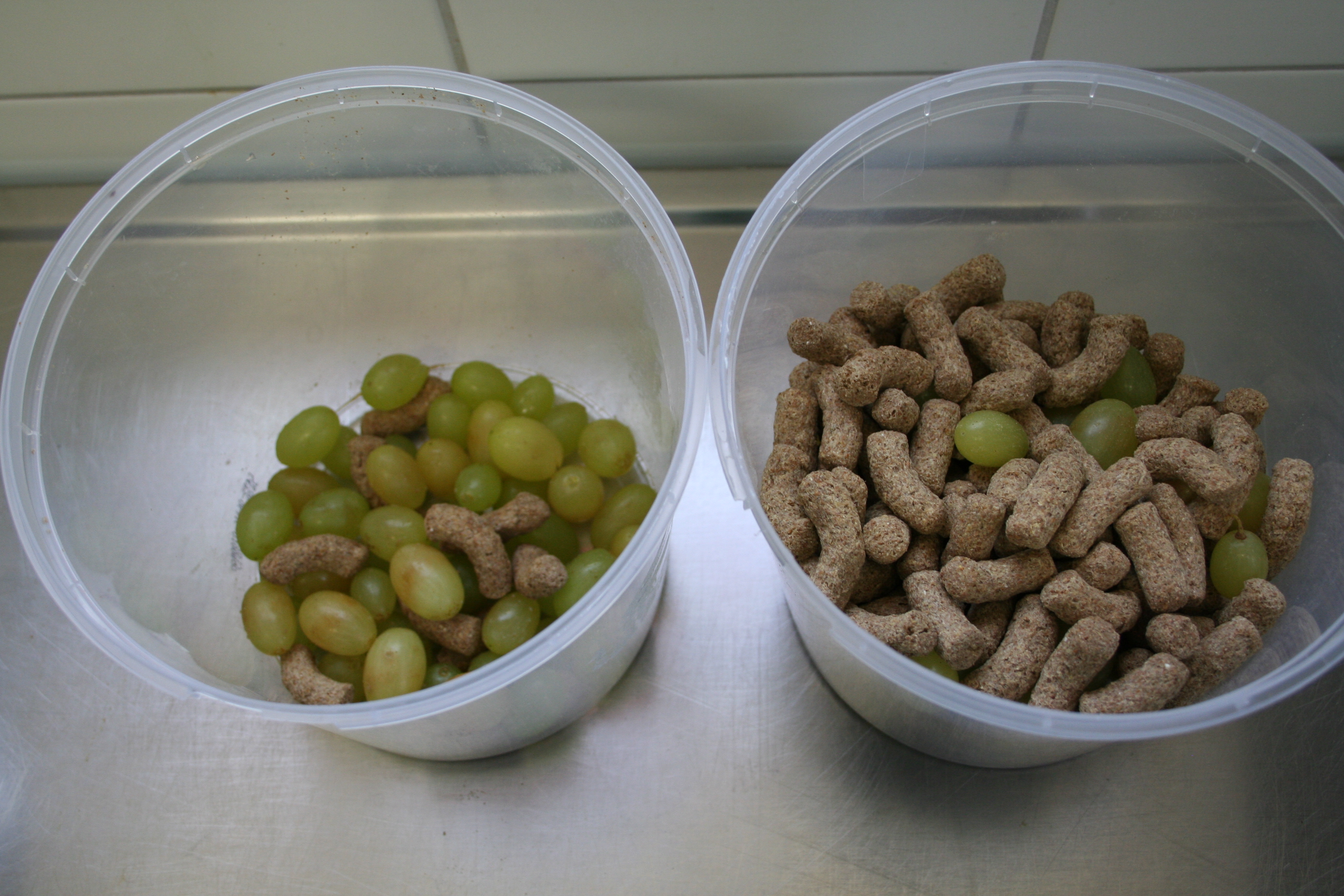

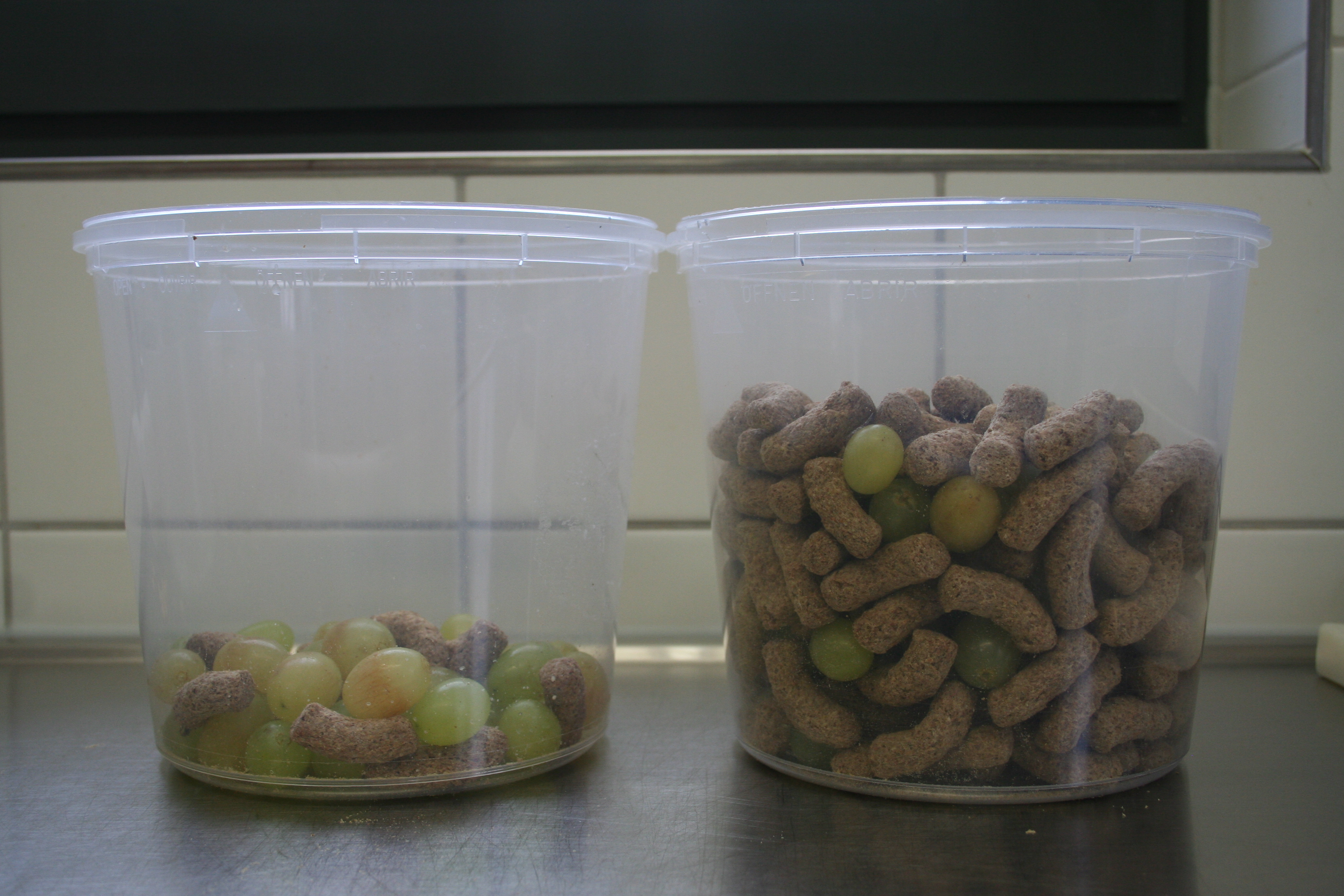
**

**Experiment 3**

**
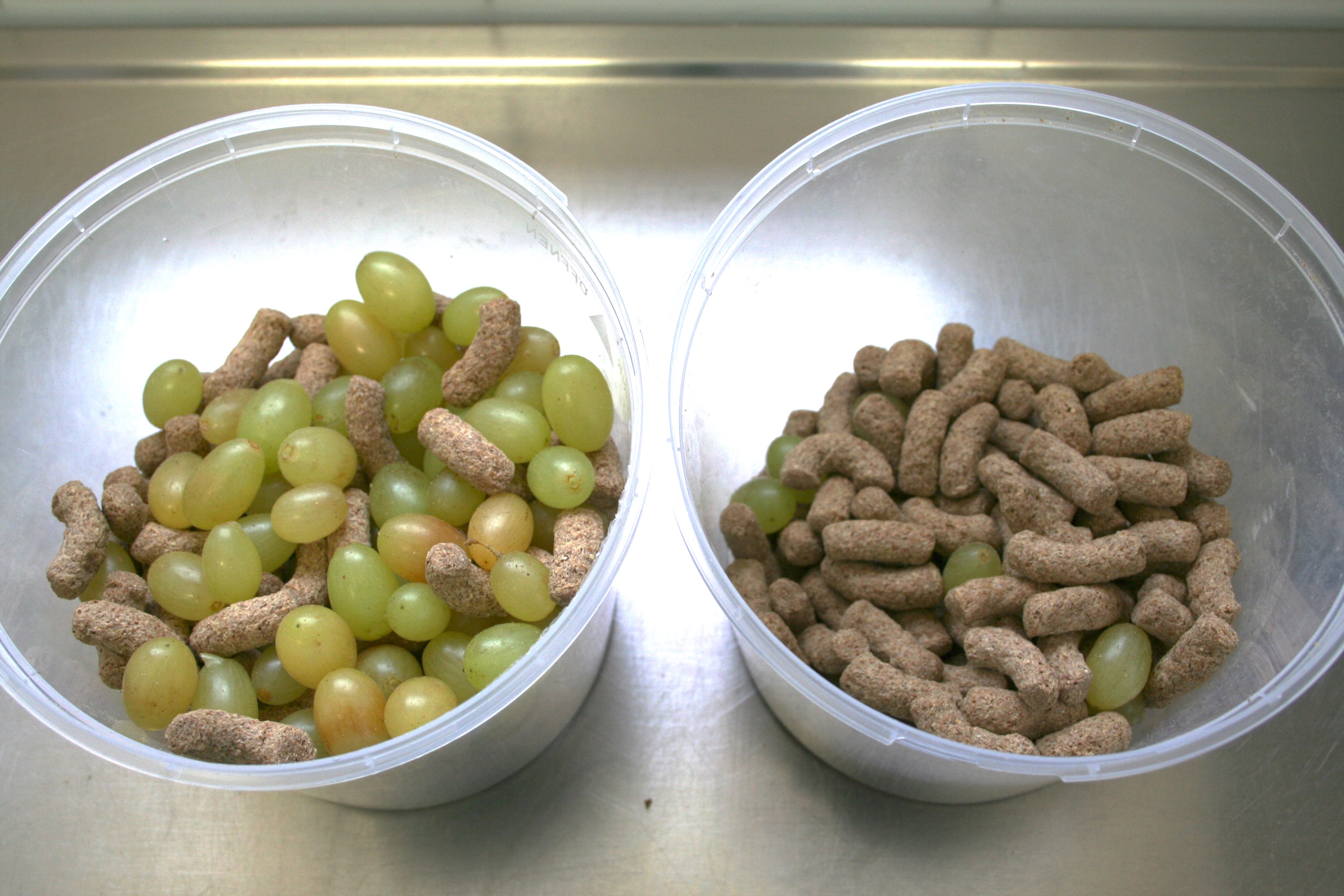

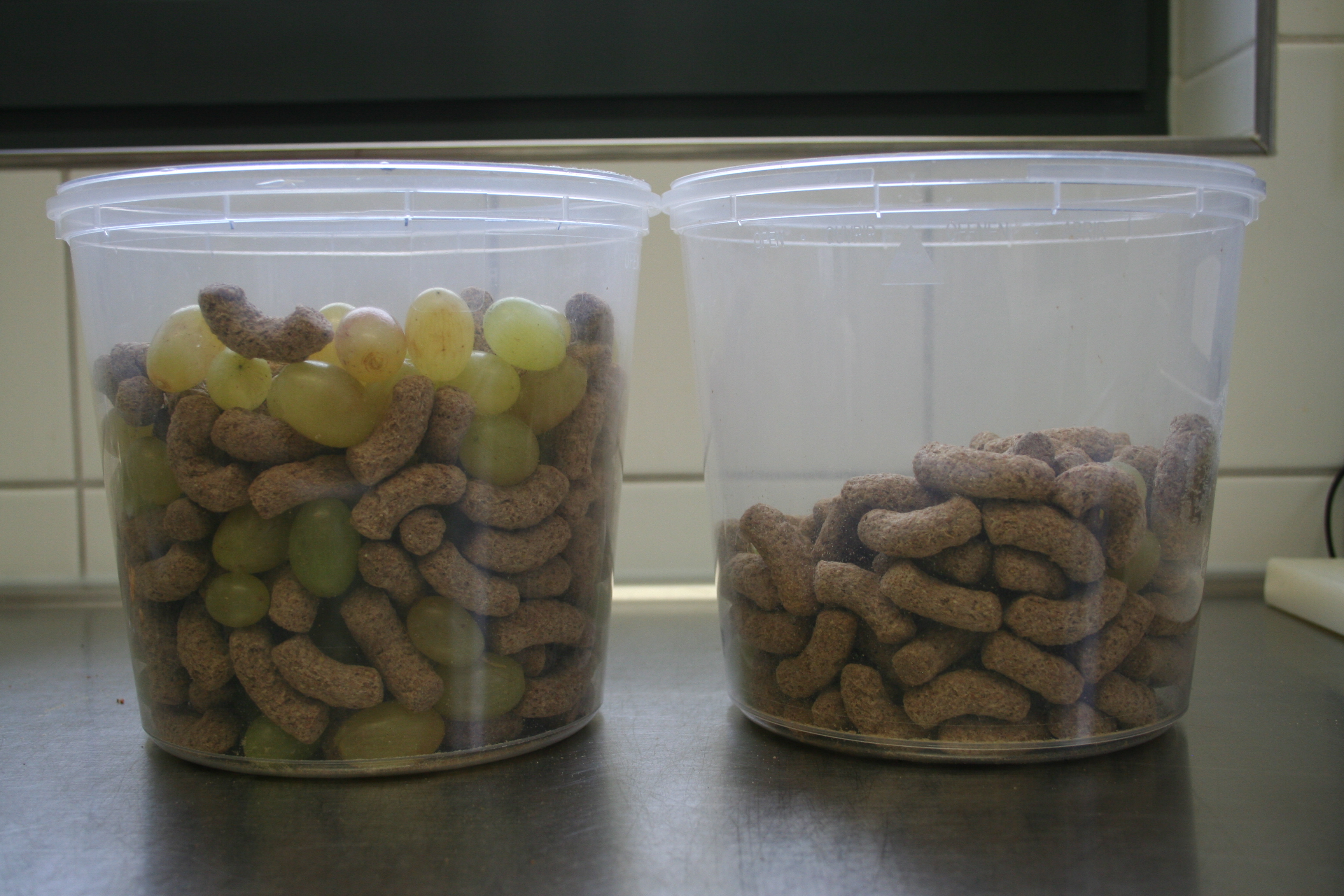
**

**Supplementary Fig. S2.** Photographs of buckets containing populations of grapes and monkey chow items that were presented to long-tailed macaques in Experiment 1a, 1b, 2a, 2b and 3. In each photograph, the favourable population is on the left.
